# Trajectories of cognitive function and associated factors in community-dwelling older adults: a prospective study

**DOI:** 10.1093/geroni/igab046.2452

**Published:** 2021-12-17

**Authors:** Zimu Wu, Robyn Woods, Elsdon Storey, Trevor Chong, Raj Shah, Suzanne Orchard, Anne Murray, Joanne Ryan

**Affiliations:** 1 Monash University, Melbourne, Victoria, Australia; 2 Monash University, Monash University, Victoria, Australia; 3 Rush, Rush University Medical Center, Illinois, United States; 4 Hennepin HealthCare Research Institute, Hennepin HealthCare, Minneapolis, Minnesota, United States

## Abstract

There is variability in cognitive aging between individuals. This study aimed to investigate cognitive aging trajectories, the associated modifiable factors, and the association of these trajectories with dementia. Community-dwelling older adults (n=19,114) without dementia or major cognitive impairment at inclusion were followed for up to 7 years, with regular standardized cognitive assessments. Group-based (multi-) trajectory modeling identified distinct cognitive trajectories. Structural equation modeling (n=16,018) was used to analyze the associated predictors. Four to seven trajectories were identified per cognitive domain, with generally stable trajectories. Improvement in verbal fluency and minor psychomotor slowing were common. Substantial decline in global cognition and episodic memory were observed in a small proportion of individuals. The highest proportions of dementia cases were in trajectories with major decline in global cognition (56.9%) and memory (33.2%). A number of sociodemographic characteristics, health behaviors and chronic conditions were either directly or indirectly associated with cognitive change in older adults. This study found that some individuals appear resilient to cognitive decline even with advancing age, and that factors that promote healthy cognitive aging are not simply the absence of factors which confer risk for decline.

